# Hemangioma of the abdominal wall in infancy

**DOI:** 10.1093/jscr/rjaf238

**Published:** 2025-04-22

**Authors:** Majd Oweidat, Mohammed Alra'e, Mosaikah Anati, Ammar W M Hassouneh, Saleh Juneidi

**Affiliations:** College of Medicine, Hebron University, Hebron, West Bank, Palestine; College of Medicine, Hebron University, Hebron, West Bank, Palestine; College of Medicine, Hebron University, Hebron, West Bank, Palestine; Department of Pediatric Surgery, Princess Alia Hebron Governmental Hospital, Hebron, West Bank, Palestine; College of Medicine, Hebron University, Hebron, West Bank, Palestine; Department of Radiology, Princess Alia Hebron Governmental Hospital, Hebron, West Bank, Palestine; College of Medicine, Hebron University, Hebron, West Bank, Palestine

**Keywords:** hemangioma, pediatric surgery, abdominal wall mass, case report

## Abstract

Hemangiomas are common benign vascular tumors in infancy, but their occurrence in the abdominal wall is rare. We report a 6-month-old female infant with a left-sided palpable abdominal wall mass. Ultrasound revealed a well-defined, vascularized soft tissue mass, but its precise location was unclear. Contrast-enhanced computed tomography showed a 4.3 × 4 × 4 cm heterogeneous mass with peripheral arterial enhancement and centripetal filling, raising suspicion for a vascular malformation or hemangioma. Magnetic resonance imaging confirmed a hypervascular mass in the lateral-posterior abdominal wall. Due to the lesion’s high vascularity, percutaneous biopsy was considered high-risk, and complete surgical excision was performed. Histopathology confirmed a benign hemangioma with organized hematoma and dystrophic calcification. The patient had an uneventful recovery, with no recurrence observed at 40-day and 6-month follow-ups. This case highlights the importance of considering hemangiomas in the differential diagnosis of abdominal wall masses in infants.

## Introduction

Hemangioma is a benign tumor of overgrown blood vessels and the most common pediatric tumor, affecting 4% to 5% of newborns [1]. Risk factors include female sex, family history, white race, placental anomalies, multiple gestations, prematurity, and low birth weight [1]. Hemangiomas are classified as congenital or infantile, with the latter being more common. Congenital hemangiomas are present at birth, while infantile hemangiomas (IH) develop later during infancy. IHs are marked by a period of rapid growth early on, followed by natural regression over time but can cause severe complications, including ulceration, necrosis, bleeding, and airway obstruction [1–3]. This case highlights a very rare incidence of hemangioma that grew in the abdominal wall of a 6-month-old infant, managed via surgical excision.

## Case report

A 6-month-old female infant was brought to medical attention directly after her grandmother noticed a palpable, left-sided solid abdominal wall mass. The family sought evaluation at an outpatient clinic, where an initial ultrasound was performed. The ultrasound identified a well-defined, heterogeneous, hypoechoic mass measuring approximately 3.7 × 3.5 cm but could not definitively determine whether the lesion originated from the abdominal wall or intra-abdominal tissues. It appeared separate from adjacent structures, including the left kidney and spleen. Color Doppler imaging demonstrated detectable peripheral arterial vascularity, though venous vascularity within the mass could not be adequately assessed. Based on these findings, the patient was referred to our tertiary care center for further evaluation and management.

Upon presentation to our department, a thorough history was obtained, and a physical examination was performed. The patient was born at 39 weeks via uncomplicated vaginal delivery with a birth weight of 3.2 kg. No prenatal ultrasound abnormalities were detected. He had been in good health with no significant prior medical or surgical history. There was no history of trauma, fever, or family history of similar conditions or malignancies. Laboratory investigations were ordered to establish a baseline profile and to rule out possible systemic involvement. Notable findings included an elevated activated partial thromboplastin time (APTT) (58.5 s; normal range: 30–40 s), decreased mean corpuscular volume (72.2 fL; normal range: 80–95 fL), and a reversed neutrophil-to-lymphocyte ratio (neutrophils 23.4%; normal range: 55%–70%; lymphocytes 63.6%; normal range: 20%–40%). The remaining laboratory results were within normal limits.

**Figure 1:**
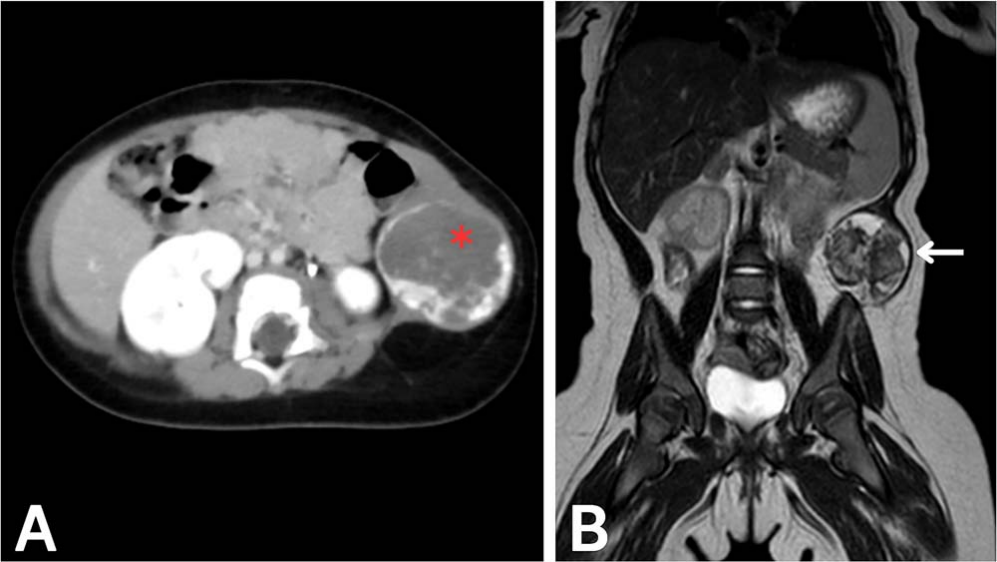
Imaging of the abdominal wall mass. (A) Axial post-contrast CT image in the venous phase demonstrates peripheral centripetal enhancement of the lesion (asterisk). (B) Coronal T2-weighted MRI reveals the anatomical relationships of the mass (arrow).

To evaluate potential intra-abdominal involvement and further characterize the mass, a contrast-enhanced computed tomography (CT) scan was performed first. As shown in Fig. 1A, the CT scan revealed a 4.3 × 4 × 4 cm round, heterogeneous mass in the left lateral abdominal wall, containing internal calcifications. The mass showed significant peripheral enhancement during the arterial phase, with centripetal filling observed in the delayed phase. Several feeding vessels were noted, the most prominent being the left inferior epigastric artery. Additional vessels were noted to arise from the internal and external iliac arteries, as well as a descending vessel from the left lateral inferior chest wall. These imaging findings were highly suggestive of a vascular malformation, although the possibility of a hemangioma could not be entirely excluded. The differential diagnosis at this stage included rhabdomyosarcoma, fibromatosis, hemangioma, and lymphatic malformation.

After CT confirmed the mass’s location in the abdominal wall, magnetic resonance imaging (MRI) was performed to delineate the mass and soft tissue involvement further. As shown in Fig. 1B, the MRI confirmed the presence of a heterointense mass in the left lateral abdominal wall, with peripheral enhancement consistent with a vascular malformation. The liver, spleen, and both kidneys appeared normal on imaging. Given the highly vascularized nature of the lesion on imaging, percutaneous biopsy was deemed high-risk; therefore, surgical excision was recommended for both diagnostic and therapeutic purposes.

Surgical excision of the mass was performed under general anesthesia. The patient was prepped and draped in a sterile fashion. A midline incision was made, and the abdominal wall was dissected layer by layer. The mass was identified in the lateral-posterior abdominal wall and completely excised. Surrounding vessels and the ureter were preserved. Hemostasis was achieved, and the surgical site was closed in layers. The patient tolerated the procedure well.

Histopathological examination demonstrated a benign vascular proliferation consistent with hemangioma. The adjacent soft tissue showed features of organized hematoma, fibrosis, and dystrophic calcification. No evidence of malignancy was identified, thereby confirming the diagnosis of hemangioma. The patient’s postoperative course was unremarkable, and she was discharged with instructions for close follow-up. At the 40-day and 6-month postoperative visits, abdominal ultrasounds were performed to assess the surgical site and to evaluate for any signs of recurrence or complications. The ultrasound revealed no evidence of recurrence or complications. The liver, gallbladder, spleen, and both kidneys appeared normal in size, shape, and echotexture, with no signs of hydronephrosis or free fluid in the abdomen.

## Discussion

Hemangiomas are commonly referred to as “strawberry marks” because of their distinctive appearance. They are caused by the proliferation of endothelial cells [3]. Hemangiomas can develop at any site in the body, and any organ may be affected. Hemangiomas are commonly found in the extremities, head, and neck, while the abdominal wall is a relatively rare site [4, 5]. The liver is the organ most frequently affected [6]. Furthermore, hemangiomas may be superficial or deep, and they can be focal, multifocal, or segmental depending on the number and anatomical locations involved [6–8]. However, hemangiomas can occasionally develop in atypical sites within the abdominal cavity, presenting with uncommon clinical features. This can cause diagnostic uncertainty, often leading to inadvertent surgical removal [6]. To the best of our knowledge, this is the first documented case of a hemangioma occurring in the lateral-posterior abdominal wall of an infant.

The exact mechanisms underlying hemangioma development are not fully understood. Multiple factors are thought to play significant roles in the pathogenesis. Notably, the primary pathogenic mechanism is abnormal angiogenesis. During this process, hemangioma-derived endothelial cells become activated, degrade the basement membrane, migrate, and proliferate to form a new vascular mass [9, 10]. Few syndromes are also associated with the hemangiomas, such as Klippel-Trénaunay syndrome and Proteus syndrome [11].

Hemangiomas grow rapidly during the first year of life and subsequently regress spontaneously. The involuted mass often leaves behind fibrofatty residuum, telangiectasia, and redundant skin [1, 2]. Despite their typically benign nature, hemangiomas can behave aggressively, causing severe and potentially life-threatening complications such as painful ulceration, necrosis, bleeding, airway obstruction, vision impairment, high-output cardiac failure, and consumptive hypothyroidism [1].

There are several treatment options available, but no standardized approach has been established so far. For symptomatic hemangiomas, pharmacological treatments such as beta-blockers, steroids, and interferons are now preferred over surgery and laser ablation. The latter are typically reserved for more complex cases [12].

In conclusion, hemangiomas should be included in the differential diagnosis of abdominal wall soft tissue masses in infants.
